# Determination of Quantum Yield in Scattering Media Using Monte Carlo Photoluminescence Cascade Simulation and Integrating Sphere Measurements

**DOI:** 10.3390/ma18153710

**Published:** 2025-08-07

**Authors:** Philip Gelbing, Joachim Jelken, Florian Foschum, Alwin Kienle

**Affiliations:** Institut für Lasertechnologien in der Medizin und Meßtechnik, Universität Ulm, Helmholtzstr. 12, 89081 Ulm, Germany

**Keywords:** quantum yield, fluorescence, luminescence, photoluminescence, monte carlo simulation, scattering media, radiative transfer equation, integrating sphere, rhodamine 6g

## Abstract

Accurate determination of the quantum yield ($\Phi_{f}$) in scattering media is essential for numerous scientific and industrial applications, but it remains challenging due to re-absorption and scattering-induced biases. In this study, we present a GPU-accelerated Monte Carlo simulation framework that solves the full fluorescence radiative transfer equation (FRTE), incorporating spectrally dependent absorption, scattering, and fluorescence cascade processes. The model accounts for re-emission shifts, energy scaling due to the Stokes shift and implements a digital optical twin of the experimental setup, including the precise description of the applied integrating sphere. Using Rhodamine 6G in both ethanol and PDMS matrices, we demonstrate the accuracy of the method by comparing simulated reflectance and transmission spectra with independent experimental measurements. $\Phi_{f}$ and emission distributions are optimized using a Levenberg–Marquardt algorithm. The obtained quantum yields agree well with literature values for Rhodamine 6G. This approach eliminates the need for empirical correction factors, enabling the reliable determination of actual, undistorted emission spectra and the $\Phi_{f}$ in complex scattering media.

## 1. Introduction

Photoluminescence materials play a central role in numerous scientific and industrial applications, including imaging, diagnostics, and DNA sequencing. In the lighting industry, they are essential for the development of white light LEDs, in which they serve as wavelength converters. This technology is characterized by high energy efficiency and longevity, whereby the quantum yield ($\Phi_{f}$) is a decisive parameter for the performance of these materials and a direct measure of luminescence efficiency [1,2]. This paper primarily discusses fluorescence, which is the rapid luminescent emission of light that occurs when fluorescent dyes, such as Rhodamine 6G, absorb photons. However, our methodological framework is applicable to various types of photoluminescence. The exact determination of the quantum yield is a complex challenge, especially in scattering media. It is influenced by various factors, including the chemical structure of the fluorophore, the ambient temperature, interactions with the solvent, and quenching processes such as internal transformations or non-radiative relaxations. Two main optical methodological approaches are used in the literature to measure the quantum yield—relative and absolute measurements [3,4,5]. In the context of relative measurement, the emission of a sample characterized by an unknown quantum yield is compared with that of a reference sample, which possesses a known quantum yield, under conditions that are identical to those of the reference sample. This method is widely employed, yet it possesses significant limitations, outlined as follows: The choice of reference materials with well characterized optical properties is limited, and matching the optical properties of the sample and the reference, especially with respect to absorption and scattering, is difficult to achieve in practice. In strongly scattering samples, re-absorption of scattered photons can significantly complicate measurements, leading to an overestimation of absorption and an underestimation of emission. Notably, re-absorption effects are not limited to scattering media. They also appear in non-scattering or weakly scattering media, particularly when high concentrations or strongly absorbing fluorophores are involved. This systematic bias can lead to an underestimation of the quantum yield. Consequently, optically thin solutions are typically employed to mitigate this issue [6,7,8,9,10]. In contrast to the relative method, absolute measurements do not necessitate a reference sample and can thus be utilized for a broader range of materials. Integrating spheres are commonly used in this context. Without the use of an integrating sphere, a spectrometer measurement would merely detect a fraction of the emitted light, as this depends on the scattering, the refractive index, the geometry of the sample, and other factors, ensuring more accurate results [3,11,12,13]. The quantum yield is then determined by comparing the total number of photons emitted to the total number of photons absorbed.

In a multitude of technical and biological systems, the processes of scattering and multiple scattering result in complex light propagation, particularly in highly scattering media such as luminescence phosphor layers or biological tissues. The interplay between absorption, fluorescence, and scattering is particularly pronounced in these contexts. The inability to dilute samples to reduce scattering often presents a significant challenge in determining the fluorescence quantum yield and the true emission spectrum in these and other scattering media.

As outlined in [14], a primary issue is that the fraction of emitted photons captured by the detector is highly dependent on the scattering and absorption properties of the samples. Accurately determining the exact proportion of photons absorbed, particularly those absorbed by the fluorophore rather than by other non-fluorescent components, is essential for measuring the correct $\Phi_{f}$. Additional complications result from inner filter effects and re-absorption processes as follows: The excitation light can undergo significant attenuation within the sample, resulting in spatially non-uniform fluorescence emission. Such spatial gradients become particularly pronounced in slab geometry, where fluorescence intensity is typically stronger on the side facing the excitation source. Furthermore, emitted photons may be re-absorbed before escaping the medium, particularly in systems with small Stokes shifts, where the absorption and emission spectra overlap significantly. These effects, along with the wavelength dependence of the medium’s optical properties, profoundly alter the light propagation within the sample. This leads to distortions in both the spectral shape and the absolute intensity of the measured fluorescence and absorption signals. Consequently, if these factors are not appropriately accounted for or corrected, the experimentally determined quantum yield may substantially deviate from the intrinsic $\Phi_{f}$ of the fluorophore.

A range of methodologies for addressing these effects can be found in the literature. In [15], an analytical approach is described to correct re-absorption effects in solid-state samples. Samples with varying thicknesses are prepared to visualize how re-absorption impacts measurements. While re-absorption effects are minimal in thin layers, they become significant in thicker layers. To ensure the accuracy of the quantum yield determination, the absorption and emission spectra of both thin and thick samples are meticulously recorded. Crucially, the approach assumes availability of an undistorted emission spectrum, typically obtained from very thin films or highly diluted solutions where re-absorption is negligible. The probability of re-absorption, denoted by *a*, is calculated using the overlap integral between absorption and emission spectra. This calculation integrates the normalized emission spectrum with the absorption coefficient based on Beer–Lambert law principles. The corrected quantum yield is subsequently calculated using the following formula:

(1) $$\Phi_{f}=\frac{\Phi_{f,\mathrm{obs}}}{1-a+a\;\Phi_{f,\mathrm{obs}}}\;.$$

A similar but extended approach is adopted in [16], where $\Phi_{f}$ of scattering media is determined using an integrating sphere under both direct and indirect illumination. The emission spectra that result from this measurement can be used as reliable indicators of undistorted emission. The same overlap-integral formalism and correction formula (Equation (1)) are then applied. This approach has been found to significantly reduce the effects of scattering and to provide more precise quantum yield measurements, especially when dealing with highly scattering media. Another approach to correcting spectral distortions by re-absorption in scattering media is described in [17]. The authors also create samples with different layer thicknesses to compare the undistorted (true) emission spectrum with the spectrum distorted by re-absorption.

While the approaches mentioned make important contributions to the determination of quantum yield in scattering media, they have specific drawbacks. They rely on *post hoc* analytical corrections based on indirect estimations of re-absorption probability rather than modeling the full propagation of light within the medium. Additionally, the requirement to prepare and analyze multiple samples with varying layer thicknesses imposes considerable experimental effort. This process is not only time consuming and resource intensive, but also introduces the potential for systematic errors, particularly in the case of inhomogeneous, very thin, or polydisperse samples.

In order to correctly determine the quantum yield under simultaneous scattering and absorption of excitation and fluorescence photons and avoid the inclusion of correction factors and sample-by-sample empirical fixes, it may be advisable to employ a physically-based model that takes into account the entire light propagation process, including absorption, scattering, and fluorescence. A promising method for describing these effects is the Fluorescent Radiative Transfer Equation (FRTE), an extension of the classical radiative transfer equation that explicitly incorporates the spectral properties of fluorescence, including wavelength shift and re-absorption [18]. Recent studies have demonstrated that the FRTE facilitates more precise modeling of light propagation in fluorescent scattering media, particularly by accounting for multiple scattering and spectrally varying absorption effects [19,20,21,22]. In this study, the FRTE is therefore employed as a foundation for the quantitative analysis of quantum yield in scattering media. In order to accurately model light propagation in fluorescence media, the utilization of commercial ray-tracing software often proves to be inadequate [21]. This is because such models typically operate in two separate steps: First, light propagation is simulated for the excitation wavelengths. Then, based on the absorbed energy and quantum yield, the emission distribution is calculated. This simplification neglects the re-absorption effects, which are especially relevant in highly fluorescent and scattering media. As a consequence, the resulting simulations can significantly deviate from experimental observations. An alternative approach was proposed in [20], which, to the best of our knowledge, constitutes the first and so far only Monte Carlo solution for broadband fluorescence systems that accounts for the full fluorescence cascade, including absorption and scattering. The model, implemented in Matlab, was validated against published transmitted-flux data. However, the computational power of this approach is limited, and it is not known whether it scales well for complex geometries and material compositions. Our current approach employs a GPU-accelerated Monte Carlo method that considers the entirety of the fluorescence cascade as well, thereby enabling the precise calculation of light propagation in complex media comprising various fluorescent, absorbing, and scattering components. Unlike previous methods, our approach involves the experimental determination and integration of specific optical properties of scattering materials, thereby ensuring a realistic simulation over a broad wavelength spectrum. A key advantage of the proposed method is the utilization of GPU parallelization, which substantially improves computational efficiency and enables simulations of full fluorescence light propagation in complex geometries over extensive excitation spectra. Another essential aspect is the direct consideration of the experimental setup in the model, particularly the integrating sphere detector, ensuring close correspondence between simulation and measurement. This integration allows precise determination of optical and fluorescent properties, such as quantum yield and wavelength-dependent emission probabilities, directly from a single measurement. Prior experimental methods frequently encountered systematic uncertainties due to inadequate modeling of complex scattering phenomena and neglected spectral overlaps between absorption and emission, issues our current methodology specifically addresses.

## 2. Theory

### 2.1. Radiative Transfer Equation

In order to understand the employed model that has been utilized, we apply the RTE for unpolarized light. This equation is widely accepted as the standard formula for describing the propagation of light in scattering media, where interference effects can be neglected. For the steady-state case at a fixed wavelength, the equation is as follows:

(2) $$\hat{s}\cdot \nabla L\left(\vec{r},\hat{s}\right)\;+\;\mu_{t}L\left(\vec{r},\;\hat{s}\right)\;=\;\mu_{s}\int L\left(\vec{r},\;{\hat{s}}^{\prime }\right)p\left(\hat{s},\;{\hat{s}}^{\prime }\right)d{\hat{s}}^{\prime }\;+\;\varepsilon \left(\vec{r},\;\hat{s}\right)\;.$$

In this context, $L(\vec{r},\hat{s})$ is defined as *radiance* at the position r in the direction of the unit vector $\hat{s}$ (per unit area and per unit solid angle). The *extinction coefficient* is defined as

(3) $$\mu_{t}=\mu_{a}+\mu_{s}\;,$$

including the *absorption coefficient* $\mu_{a}$ and the *scattering coefficient* $\mu_{s}$. The *phase function* $p(\hat{s},{\hat{s}}^{\prime })$ indicates how light is redistributed from the direction ${\hat{s}}^{\prime }$ into the direction $\hat{s}$. The term $\varepsilon (r,\hat{s})$ denotes additional radiation sources [23]. When fluorescence is considered, it is imperative to extend the RTE to account for the spectral dependence of the optical properties. This entails consideration of the entire wavelength spectrum rather than a single wavelength only. The extended *FRTE* is expressed as follows:

(4) $$\begin{array}{cc} \; & \hat{s}\cdot \nabla L\left(\lambda ,r,\hat{s}\right)+\mu_{t}\left(\lambda \right)L\left(\lambda ,r,\hat{s}\right)=\\ \; & \;\mu_{s}\left(\lambda \right)\int_{4\pi }L\left(\lambda ,r,{\hat{s}}^{\prime }\right)\;p\left(\lambda ,\hat{s},{\hat{s}}^{\prime }\right)\;d\Omega^{\prime }+\varepsilon \left(\lambda ,r,\hat{s}\right)\\ \; & \;+\frac{1}{4\pi }\Phi_{f}\int \mu_{a,f}\left(\lambda^{\prime }\right)\left[\int_{4\pi }L\left(\lambda^{\prime },r,{\hat{s}}^{\prime }\right)\;d\Omega^{\prime }\right]P_{f}\left(\lambda \right)\;d\lambda^{\prime }\;. \end{array}$$

The additional term represents the secondary light source generated by fluorescence within the media. The absorption of the fluorophore at wavelength $\lambda^{\prime }$ is quantified by the spectral absorption profile $\mu_{a,f}\left(\lambda^{\prime }\right)$. The radiation at emission wavelength λ is assumed to be isotropically emitted. The quantum yield $\Phi_{f}$ is a material-dependent constant, as it describes the ratio of emitted to absorbed photons:

(5) $$\Phi_{f}=\frac{N_{\mathrm{em}}}{N_{\mathrm{abs}}}\;.$$

The energy transfer processes within fluorescent molecules can be effectively visualized using the Jablonski diagram [1,24]. Following photon absorption, a molecule is excited into a higher vibronic state of an electronic level (e.g., $S_{1}$ or $S_{2}$). According to the Franck–Condon principle [25,26], these transitions occur vertically in the Jablonski diagram due to the disparity between rapid electronic excitation and slower nuclear motion. As a result, molecular geometry remains unchanged during excitation. Subsequently, the molecule undergoes ultrafast internal conversion, typically within femto- to picoseconds. This process dissipates excess vibrational energy non-radiatively to the environment, leading to relaxation of the molecule to the lowest vibronic level of $S_{1}$. By definition, this non-radiative relaxation occurs without photon emission. Fluorescence emission then takes place through the radiative transition from the lowest excited singlet state ($S_{1}$) to the ground electronic state ($S_{0}$), accompanied by the emission of a photon. This behavior is encapsulated by the *Kasha rule* [27], which states that fluorescence predominantly arises from the lowest excited singlet state ($S_{1}$), regardless of the initially excited electronic state (e.g., $S_{2}$ or higher). As fluorescence emission consistently occurs from the same initial state ($S_{1}$) and is predominantly determined by molecular structure, the quantum yield $\Phi_{f}$ is effectively independent of the excitation wavelength $\lambda^{\prime }$. In the absence of additional decay pathways, such as non-radiative quenching or energy transfer mechanisms, the fluorescence quantum yield remains constant for a given material, irrespective of the specific excitation wavelength [1]. The spectral distribution of emitted photons is characterized by a normalized probability density function (PDF), denoted as ${\tilde{P}}_{f}\left(\lambda \right)$, satisfying the following normalization condition:

(6) $$\int_{0}^{\infty }{\tilde{P}}_{f}\left(\lambda \right)\;d\lambda =1\;.$$

Due to refraction, scattering, and re-absorption effects within the medium, this probability distribution does not directly correspond to the experimentally measured emission spectrum. Instead, it represents the intrinsic probability distribution for the emission of a single photon from an individual fluorescence event.

### 2.2. Monte Carlo Implementation

The FRTE (4) is solved using a GPU-accelerated Monte Carlo simulation. The GPU kernel is implemented in OpenCL, while host-side control, data preprocessing, and postprocessing are handled via Python (version 3.13) and ISO C++ 14-Standard. All simulations were performed on a workstation equipped with an AMD Ryzen 9 7900X CPU (12 cores, 4.7 GHz; Advanced Micro Devices, Santa Clara, CA, USA) and a single NVIDIA GeForce RTX 4070 Ti GPU (NVIDIA Corporation, Santa Clara, CA, USA), using CUDA version 12.3. For each of the *N* simulated photons, pseudo-random numbers (PRNs) determine the initial position, propagation angle, and wavelength. The starting wavelengths are sampled from the experimentally measured spectrum, incorporating the hyperchromator calibration and spectrometer instrumental response (see Section 2.4). The optical properties ($\mu_{s}$, $\mu_{a}$, phase function, and refractive index) are assigned for both non-fluorescent and fluorescent components at each wavelength. For each material, these are determined prior to simulation using the method developed by us and described in [28]. The optical properties of each material are represented by the weighted contributions of its constituent components, which may include fluorescent and non-fluorescent fractions. Each component retains a distinct set of wavelength-dependent optical properties. At each step in the simulation, the probability of absorption versus scattering is calculated. The decision process for each photon is summarized in Algorithm 1, which calls for either the absorption (Algorithm 2) or the scattering (Algorithm 3) subroutine, depending on the outcome.
**Algorithm 1** Photon interaction: absorption vs. scattering**Require:** Photon with wavelength λ and weight $w_{\lambda }$**Require:** Absorption: $\mu_{a}^{\mathrm{base}}\left(\lambda \right)$, $\mu_{a}^{\mathrm{fluo}}\left[i\right]\left(\lambda \right)$
**Require:** Scattering coefficient $\mu_{s}^{\mathrm{tot}}\left(\lambda \right)$
**Require:** Random number ξ∼U[0,1)  1:Compute total absorption: $\mu_{a}^{\mathrm{tot}}=\mu_{a}^{\mathrm{base}}+\sum_{i}\mu_{a}^{\mathrm{fluo}}\left[i\right]$  2:Compute absorption probability: $P_{\mathrm{abs}}=\mu_{a}^{\mathrm{tot}}/\left(\mu_{a}^{\mathrm{tot}}+\mu_{s}^{\mathrm{tot}}\right)$  3:Draw random number ξ  4:**if** $\xi <P_{\mathrm{abs}}$ **then**  5:    **Call Algorithm 2**: Absorption  6:**else**  7:    **Call Algorithm 3**: Wavelength-Dependent Scattering  8:**end if**

**Algorithm 2** Absorption with fluorescence and isotropic re-emission**Require:** Quantum yield $\Phi_{f}\left[i\right]$, emission CDF[i](λ)
**Require:** Random numbers $\xi_{0},\xi_{1},\xi_{2}∼U\left[0,1\right)$

    1:Draw $\xi_{0}\in \left[0,1\right)$    2:**if** $\xi_{0}<\mu_{a}^{\mathrm{base}}/\mu_{a}^{\mathrm{tot}}$ **then**    3:    Photon is absorbed in non-fluorescent base material (no re-emission)    4:
**else**
    5:    **for** each fluorescent component *i* **do**    6:        Accumulate: $\mu_{a}^{\mathrm{cum}}+=\mu_{a}^{\mathrm{fluo}}\left[i\right]$    7:        **if** $\xi_{0}<\mu_{a}^{\mathrm{cum}}/\mu_{a}^{\mathrm{tot}}$ **then**    8:           Draw $\xi_{1}\in \left[0,1\right)$    9:           **if** $\xi_{1}>\Phi_{f}\left[i\right]$ **then**  10:               Photon is absorbed in component [i] (no re-emission)  11:           **else**  12:               Photon is re-emitted  13:               Update: $\lambda \leftarrow \lambda^{\prime }$, $w_{\lambda }\leftarrow w_{\lambda^{\prime }}\cdot \frac{\lambda^{\prime }}{\lambda }$  14:               Emit isotropically:  15:               Draw $\xi_{1},\xi_{2}\in \left[0,1\right)$  16:               Compute: $\cos\theta =1-2\xi_{1}$,    $\phi =2\pi \xi_{2}$  17:               Set direction: ${\vec{v}}_{\mathrm{photon}}\leftarrow \left(\sin\theta \cos\phi ,\sin\theta \sin\phi ,\cos\theta \right)$  18:           **end if**  19:           **break**  20:        **end if**  21:    **end for**  22:
**end if**



Photons propagate through the medium until absorption or interaction with a boundary occurs [23]. Absorption is treated distinctly for fluorescent and non-fluorescent components. The total absorption coefficient is defined as the sum of all component absorptions, and the component in which absorption occurs is probabilistically determined according to the relative contributions of the components. A PRN is used to select the absorbing component (Algorithm 2). If the photon is absorbed in a non-fluorescent region, it is terminated. If absorbed in a fluorescent component, re-emission is determined probabilistically using $\Phi_{f}$. If another PRN exceeds the quantum yield, the photon is terminated; in all other cases, it is re-emitted with a new wavelength. To model the intrinsic emission probability ${\tilde{P}}_{f}\left(\lambda \right)$ introduced in Equation (6), we employ a normalized Gaussian mixture model (GMM) with three components. Each component represents a Gaussian distribution defined by its mean $\mu_{i}$, standard deviation $\sigma_{i}$, and weight $w_{i}$, as follows:

(7) $${\tilde{P}}_{f}\left(\lambda \right)\approx \sum_{i=1}^{3}w_{i}\cdot N\left(\lambda |\mu_{i},\sigma_{i}^{2}\right)\;,\;\mathrm{with}\;\sum_{i=1}^{3}w_{i}=1\;.$$

To enforce normalization, only two of the weights are treated as free parameters, with the third defined as $w_{3}=1-w_{1}-w_{2}$. The model is therefore parameterized by eight independent variables as follows: three means $\mu_{i}$, three standard deviations $\sigma_{i}$, and two weights. The cumulative distribution function (CDF) associated with ${\tilde{P}}_{f}\left(\lambda \right)$ is used to generate random emission wavelengths during the Monte Carlo sampling process. However, the true probability distribution of re-emitted wavelengths is not directly accessible. Due to multiple cycles of re-absorption and re-emission, the effective emission spectrum differs from the initially measured fluorescence spectrum. As a pragmatic starting point, the GMM parameters are initialized by fitting the model to the experimentally measured fluorescence spectrum, which serves as a proxy for the intrinsic emission probability distribution. These initial parameters are subsequently refined by comparing the results from a complete Monte Carlo simulation, accounting for the full complexity of light propagation in the sample, with experimental measurements. Initially, we explored other models, such as cubic spline models, due to their ability to flexibly represent complex emission spectra, which may be necessary for other luminescent materials. However, the increased number of fitting parameters and their interdependencies reduced numerical robustness and computational efficiency during optimization.

The assignment of a new wavelength upon re-emission is achieved by sampling a PRN from the CDF. Each time a photon is re-emitted at a different wavelength, its energy is modified accordingly. This energy change can be handled in two ways as follows: either by retrospectively computing the ratio of final to initial wavelengths during postprocessing, or by continuously propagating a photon weight variable throughout the simulation. The latter method, which is adopted here, enables the use of a broad excitation spectrum and allows direct normalization to the total number of incident photons. To account for the shift in energy, the photon weight is updated according to

(8) $$w_{\lambda }=w_{\lambda^{\prime }}\left(\frac{\lambda^{\prime }}{\lambda }\right),$$

where $w_{\lambda^{\prime }}$ and $\lambda^{\prime }$ denote the photon’s weight and wavelength prior to emission, and $w_{\lambda }$ and λ after emission. If neither absorption or emission occurs, the photon undergoes scattering.

In materials such as Rhodamine 6G, where the fluorophore itself exhibits negligible scattering, the new propagation direction is determined by the phase function of the non-fluorescent base material. In heterogeneous media composed of multiple components with distinct scattering coefficients, the respective phase function is selected according to their relative contribution to the total scattering. Our system exhibits strong forward scattering. The Henyey–Greenstein phase function was selected due to its computational efficiency and analytical tractability when sampling scattering angles. It supports wavelength-dependent anisotropy g(λ) and is therefore compatible with spectrally resolved light propagation in fluorescent materials. Alternative phase functions may be implemented for strongly anisotropic or size-dependent scattering, but these were not required for the present system. If a photon exits the media, it may reach the virtual integrating sphere detector either in forward (transmission) or backward (reflectance) directory, depending on its trajectory and angular distribution. In the present study, we assume a slab geometry for all samples, where the forward and backward directions correspond to transmission and reflectance, respectively. By modeling the individual components of a sample separately in the Monte Carlo simulation, a more detailed representation of photon propagation becomes possible. This allows for the isolated study of fluorescent and non-fluorescent contributions to the measured signal. In theory, this approach enables the estimation of the quantum yield of complex material combinations. Materials such as Rhodamine contribute minimally to scattering, making the scattering coefficient of added scatterers the dominant factor. In contrast, other luminescent materials may exhibit strong intrinsic scattering. This explicit modeling allows complex cascades of light propagation to be simulated, for example in the context of optimizing phosphor-converted white LEDs.
**Algorithm 3** Wavelength-dependent scattering**Require:** Scattering coefficients $\mu_{s}^{j}\left(\lambda \right)$ for each material component *j*
**Require:**
Phase function (e.g. Henyey–Greenstein) $p_{j}\left(\theta ;g_{j}\left(\lambda \right)\right)$ for each *j*
**Require:**
Random number ξ∼U[0,1)    1:Compute total scattering coefficient: $\mu_{s}^{\mathrm{tot}}=\sum_{j}\mu_{s}^{j}\left(\lambda \right)$    2:Draw ξ∈[0,1)    3:Initialize cumulative scattering: $\mu_{s}^{\mathrm{cum}}\leftarrow 0$    4:**for** each scattering component *j* **do**    5:    Update cumulative scattering: $\mu_{s}^{\mathrm{cum}}+=\mu_{s}^{j}\left(\lambda \right)$    6:    **if** $\xi <\mu_{s}^{\mathrm{cum}}/\mu_{s}^{\mathrm{tot}}$ **then**    7:        Sample scattering angle θ from phase function $p_{j}\left(\theta ;g_{j}\left(\lambda \right)\right)$    8:        **break**    9:    **end if**  10:**end for**

### 2.3. Integrating Sphere Model

**Figure 1:**
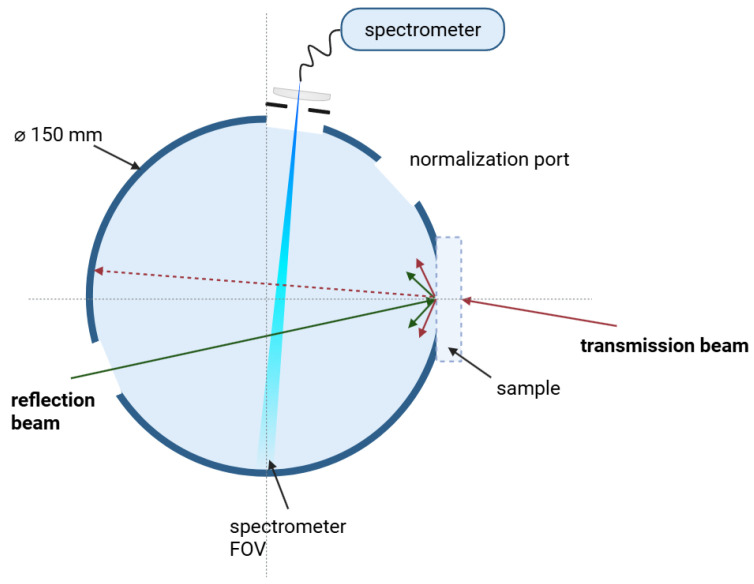
Schematic of the integrating sphere setup used in the measurement. The sample is mounted externally, and photons are either reflected back into the sphere or transmitted through the sample.

To accurately compare the measurement with the Monte Carlo simulation, the specific characteristics of the experimental environment must be taken into account. Photons emerging from a sample slab, either in transmission or reflectance, can enter the integrating sphere depending on their exit angle, position, and the sphere’s geometry. The measurement setup, as delineated in [29], utilizes a 150 mm diameter integrating sphere. The integrating sphere is coupled to a spectrometer (HR6 XR, OceanOptics, Orlando, FL, USA) with a 2048-pixel CCD array. Equipped with a 300 lines/mm grating and a 100 µm entrance slit, it has an optical resolution of 3.7 nm over a 200–1100 nm spectral range. The detector exhibits a dynamic range of approximately 12,000:1. To calibrate the system response, including the integrating sphere, the coupling optics and spectrometer, a broadband reference source with known output spectrum was measured using a power meter. This enabled the conversion of measured counts to radiant power. Detector linearity was independently verified in the relevant intensity range and found to be sufficient. In order to minimize the influence of the sample on the internal mixing process, the sample is mounted externally to the sphere. Excitation for reflectance measurements is achieved through an opposing port, thereby ensuring clear separation between transmitted and reflected light, thus reducing systematic errors. A schematic overview of the setup is shown in Figure 1, highlighting the external sample placement and separation of the transmission and reflection beams. The inner surface of the sphere is coated with barium sulfate, and the setup contains four ports, as follows: a reflection port, a normalization port, a sample port, and a detector port. The use of a baffle is avoided, as this has been shown to disrupt the internal mixing process within the sphere. However, this configuration allows for the direct illumination of the detector field of view (FOV) by photons reflected by the sample, emitted or re-emitted due to fluorescence. A proportion of the emitted light may also escape through one of the open ports, contributing no signal, while the remaining photons interact with the inner sphere wall. The reflectance of the photon is determined by the coating’s properties, and the photons undergo multiple diffuse reflections within the sphere. Consequently, the simulation must account for the spatial and angular distribution of energy that reaches various components of the sphere, depending on the wavelength and optical properties of the sample. The final fraction of light detected by the spectrometer is computed using the following analytically derived model, which is extended to incorporate spectral dependence, based on [29]

(9) $$\Phi_{D}\left(\lambda \right)=\Phi_{D0}\left(\lambda \right)+\frac{A_{\det}}{A_{\mathrm{sph}}}\sum_{n=1}^{N}\rho_{n}\left(\lambda \right)\Phi_{n}\left(\lambda \right){\left(1-\sum_{l=1}^{N}\rho_{l}\left(\lambda \right)\frac{A_{l}}{A_{\mathrm{sph}}}\right)}^{-1}\;.$$

In this context, the symbol $\Phi_{D0}\left(\lambda \right)$ denotes the fraction of energy at wavelength λ that directly reaches the detector FOV without internal scattering inside the sphere. The second term accounts for the geometric series of reflected contributions from all the inner sphere surfaces *n*, including the ports and the sample. Each surface *n* is characterized by its area $A_{n}$ and wavelength-dependent reflectance $\rho_{n}\left(\lambda \right)$, and it is irradiated by radiant power $\Phi_{n}\left(\lambda \right)$. The value of $\Phi_{n}\left(\lambda \right)$ can be determined by taking into account the angular distribution of the light entering the integrating sphere. For light scattered and emitted by the sample, this angular distribution is obtained from the Monte Carlo simulation described in Section 2.2. The open ports are modeled with ρ=0. To determine the spectral reflectance of the sample, an auxiliary Monte Carlo simulation was conducted with the same optical properties as in the initial simulation. Due to the geometry of the inner sphere and the diffuse mixing process within it, the sample is reached by photons with a quasi-isotropic angular distribution, which can be well approximated by a cosine-distributed source [29].

### 2.4. Source Model

To correctly determine the excitation spectrum $\Phi_{s}\left(\lambda \right)$ incident on the sample during measurement, it is important to recognize that the spectrum that is recorded by the spectrometer, $\Phi_{D}\left(\lambda \right)$, does not directly represent the true source spectrum prior to illumination. Several correction steps must therefore be applied to account for the specific properties of the measurement setup. First, the reflectance of the integrating sphere wall as a function of wavelength, $\rho_{\mathrm{wall}}\left(\lambda \right)$, was experimentally determined from calibration measurements at each wavelength. Assuming an open-port configuration with no direct excitation of the detector field of view (i.e., $\Phi_{D0}\left(\lambda \right)=0$), the effective source intensity in transmission geometry was obtained by inverting the integrating sphere model from Equation (9) to

(10) $$\Phi_{s}\left(\lambda \right)=\frac{\Phi_{D}\left(\lambda \right)\cdot A_{\mathrm{sph}}\cdot \left(1-\rho_{\mathrm{wall}}\left(\lambda \right)\cdot \frac{A_{\mathrm{wall}}}{A_{\mathrm{sph}}}\right)}{A_{\det}\cdot \rho_{\mathrm{wall}}\left(\lambda \right)}\;.$$

In the case of reflectance measurements, where the excitation light is redirected onto the sample by means of a reflective mirror, the mirror’s reflectance, $\rho_{\mathrm{mirror}}\left(\lambda \right)$, is included. The effective excitation spectrum $\Phi_{s}^{\mathrm{rem}}\left(\lambda \right)$ at the sample position is then given by

(11) $$\Phi_{s}^{\mathrm{rem}}\left(\lambda \right)=\frac{\Phi_{D}\left(\lambda \right)\cdot \frac{A_{\mathrm{sph}}}{A_{\det}}\cdot \left(1-\left[\rho_{\mathrm{wall}}\left(\lambda \right)\cdot \frac{A_{\mathrm{wall}}}{A_{\mathrm{sph}}}+\rho_{\mathrm{mirror}}\left(\lambda \right)\cdot \frac{A_{\mathrm{mirror}}}{A_{\mathrm{sph}}}\right]\right)}{\rho_{\mathrm{mirror}}\left(\lambda \right)}\;.$$

All measured spectra were normalized to a common integration time to account for wavelength-dependent acquisition times used to optimize the signal-to-noise ratio. The finite spectral resolution of the detection system broadens the recorded spectrum relative to the physical illumination spectrum. The spectrometer’s instrument function was independently measured using a reference light source with a negligible intrinsic linewidth. This instrument function was then used to numerically deconvolve the measured spectrum. The reconstructed excitation function, $\Phi_{s}\left(\lambda \right)$, is used in the Monte Carlo simulation to sample the initial wavelengths at the start of each photon trajectory, as well as the total intensity of the source. This ensures consistency with the spectral resolved excitation conditions of the experimental setup.

### 2.5. Sample Preparation and Reference Dye

As a reference dye, we used *Rhodamine 6G (CAS 989-38-8)* supplied by AAT Bioquest, Inc. For the experimental determination of $\Phi_{f}$, we dissolved Rhodamine 6G in ethanol at different concentrations. Three solutions were prepared as follows: $c_{1}=3.8\;\mu M$, $c_{2}=7.9\;\mu M$, and $c_{3}=15.4\;\mu M$, corresponding to a simple scaling where $c_{3}\approx 2c_{2}\approx 4c_{1}$. Since the dye itself exhibits negligible scattering, we added 1% polystyrene scatterers with a 2.4 µm diameter to the solution to introduce controlled scattering conditions. The solutions were poured into custom-made cuvettes consisting of two 1 mm-thick BK7 glass plates, which were separated by a 4 mm spacer. After filling, the cuvettes were sealed with Parafilm to prevent evaporation. In the simulation, the refractive indices of both the BK7 glass and the surrounding air were taken into account. In addition to the liquid-phase samples, we also investigated Rhodamine 6G embedded in a solid matrix. For this, a Rhodamine-ethanol solution was mixed into a *polydimethylsiloxane (PDMS)* matrix (Elastosil M 4641 A/B, Wacker Chemie AG, Munich, Germany), together with *zirconium doxide (${\text{ZrO}}_{2}$)* scatterers (diameter: 800 nm; US Research Nanomaterials Inc., Houston, TX, USA), resulting in a reduced scattering coefficient $\mu_{s}^{\prime }\approx 12$ ${\text{mm}}^{-1}$. Two dye concentrations were prepared as follows: $c_{a}=4.7\;\mu M$ and $c_{b}=2.35\;\mu M$. In the ethanol-based solutions, polystyrene particles were preferred over ${\text{ZrO}}_{2}$, due to their slower sedimentation kinetics, which ensures a more homogeneous distribution throughout the measurement duration, including optical property characterization. After determining the optical properties of the samples [28], the fluorescence quantum yield $\Phi_{f}$ and the parameters of the emission PDF, modeled as a GMM, were optimized. This was achieved by fitting Monte Carlo simulations to the experimentally recorded reflectance and transmission spectra using a Levenberg–Marquardt algorithm [30]. The optimization minimized the root-mean-square error (RMSE) between simulated and measured intensity of the sample.

## 3. Results

### 3.1. Quantum Yield in Ethanol

**Figure 2:**
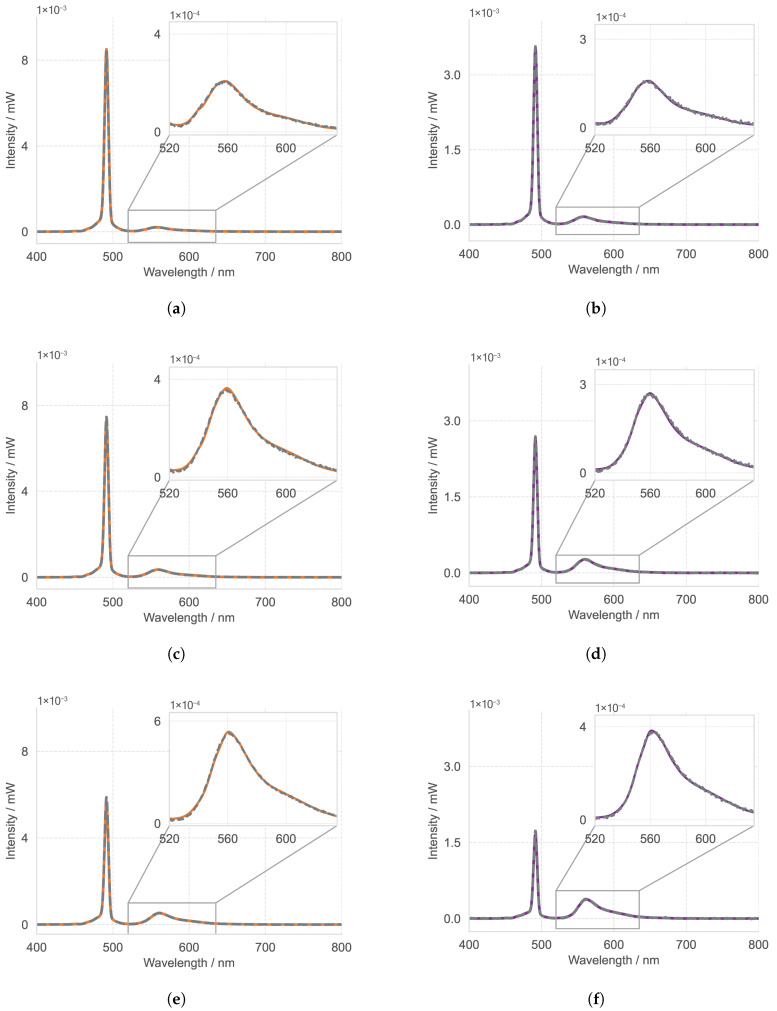
Reflectance (**left**) and Transmission (**right**) spectra for three concentrations $c_{1}$, $c_{2}$, and $c_{3}$. Subfigures are arranged by concentration from top to bottom and labeled as (**a**) $c_{1}$ reflectance, (**b**) $c_{1}$ transmission, (**c**) $c_{2}$ reflectance, (**d**) $c_{2}$ transmission, (**e**) $c_{3}$ reflectance, and (**f**) $c_{3}$ transmission. The gray dashed line represents the experimental measurement, the orange solid line corresponds to the simulated reflectance, and the purple solid line corresponds to the simulated transmission.

Figure 2 and Table 1 show the fitted spectra and quantum yields for the three Rhodamine 6G concentrations. The statistical uncertainty is derived from the square root of the diagonal elements of the covariance matrix obtained in the Levenberg–Marquardt optimization. The excitation peak, visible in both transmission and reflectance spectra in Figure 2, decreases with increasing dye concentration. This is attributed to stronger absorption of the excitation light and thus enhanced fluorescence emission. The spectral shape and position of this peak are determined by the optical properties of the sample. Additional contributing factors include the integrating sphere model and the calibrated source spectrum. Notably, these are independent of the fitted $\Phi_{f}$ or the emission PDF. Therefore, deviations between the simulation and measurement in the relative heights of the excitation peaks can be indicative of errors in the optical properties: for instance, an overestimated scattering coefficient would decrease the transmission peak while increasing the reflectance signal. This effect is analogous to that of an incorrect sample thickness. According to the light propagation model [29], an error in the sample thickness translates roughly linearly into an error in the retrieved optical parameters (e.g., a 5% thickness error propagates almost linearly to ≈5% in $\mu_{s}$ and $\mu_{a}$). This, in turn, affects the predicted fluorescence and excitation characteristics. Hence, the excitation peak serves as an internal consistency check, unaffected by the outcome of the optimization. In contrast, the magnitude of the fluorescence signal is highly sensitive to the value of $\Phi_{f}$. An overestimated $\Phi_{f}$ would lead to an excess emission in both transmission and reflectance spectra, while an underestimated $\Phi_{f}$ to lack of emission. All values are reported with two decimal places.

Published quantum yields for Rhodamine 6G in ethanol vary depending on method and concentration, but typically fall within the range of 0.89–0.95 [3,5,31,32,33,34]. It should be emphasized that these reference values are based on low-scattering systems or pure solutions. Our method, in contrast, allows for the retrieval of quantum yield even in the presence of significant scattering. This was made possible by explicitly modeling the scattering contribution of the added microspheres and simulating complex light propagation in layered or heterogeneous samples. Würth et al. [33] reported values of 0.92±0.05 using an absolute method based on a calibrated integrating sphere. Their values are concentration-dependent and corrected for re-absorption. They also fall within the range of our results. Overall, the retrieved values of $\Phi_{f}=0.91$, 0.94, and 0.93 are within their respective error ranges, demonstrating agreement with the literature. Nevertheless, differences between individual measurements warrant further analysis. Empirical evidence indicates that the quantum yield for Rhodamine 6G is concentration-dependent [31,35,36]. Lower dye concentrations might also present higher uncertainties due to weaker emission signals and sensitivity to calibration errors. Larger relative errors can be observed in conditions with lower absorption ($\mu_{a}$), such as in sample $c_{1}$, and scattering ($\mu_{s}$) coefficients [29]. Variations between assumed model parameters and actual physical sample conditions, or errors in detector sensitivity calibration across wavelengths, can impact accuracy, especially for small optical thicknesses. Quantum efficiency can also exhibit temperature dependence even for small changes, typically decreasing with increasing temperatures [37]. Temperature fluctuations during measurements or differences from the literature conditions may therefore contribute additional variance to the quantum yields.

### 3.2. Effect of Re-Absorption on the Quantum Yield

Conventional absolute integrating sphere protocols estimate $\Phi_{f}$ by dividing the detected emission by the total number of *all primary* absorption events of the sample. This approach is valid only for dilute, non-scattering samples in which the absorption and emission spectra have negligible overlap. However, in practical scenarios, additional mechanisms can bias the determination of $\Phi_{f}$.

One such mechanism is the re-absorption of fluorescence photons when emission and absorption bands overlap. Photons initially emitted via fluorescence can be re-absorbed and re-emitted multiple times before escaping the sample. Each absorption-emission cycle typically red shifts the spectrum, increasing the probability of loss through non-radiative decay pathways, thereby reducing the detected emission. The probability of these cascading absorption–emission events increases with optical thickness and is further amplified by multiple scattering. As a result, the measured fluorescence underestimates the true amount of photons generated by the initial excitation events.

This effect is captured by our simulations, which distinguish between photons absorbed at the excitation wavelength and subsequent fluorescent photons, while tracking the history of absorption–emission cycles. If we quantify all fluorescent photons exiting the sample via either reflectance or transmission and divide by the number of primary absorption events, we obtain an uncorrected quantum yield of $\Phi_{f}^{\mathrm{uncorr}}=0.87$ for concentration $c_{3}$. This contrasts with the intrinsic quantum yield obtained from our simulation, $\Phi_{f}^{\mathrm{true}}=0.93$.

**Figure 3:**
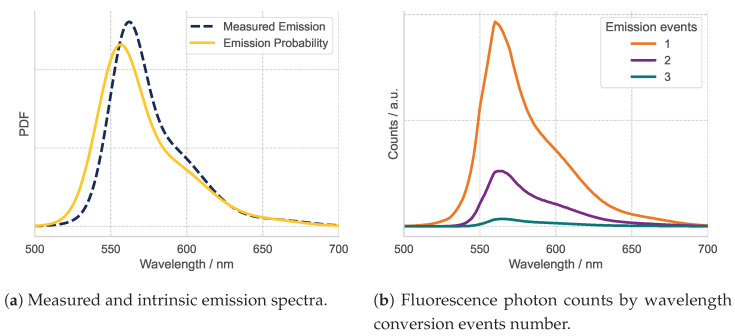
(**a**) Normalized emission spectra: the measured spectrum (recorded using an integrating sphere setup) is compared to the intrinsic emission PDF used in the simulation. Both are normalized to unit area for comparability. (**b**) Distribution of simulated fluorescence photon counts in reflectance, grouped by the number of emission (wavelength conversion) events each photon underwent. Data shown for sample $c_{3}$.

Figure 3a illustrates this discrepancy. The blue dashed curve represents the initial GMM fit to the measured reflectance spectrum of the $c_{3}$ sample. The solid yellow curve shows the final fitted emission probability distribution used in the Monte Carlo simulations to assign photon wavelengths after each emission event. A distinct ∼5 nm blue shift and an increased probability at shorter wavelengths is observed compared to the measured spectrum. This reflects the cumulative effect of re-absorption events, which favors the survival of photons having a shorter wavelength. Figure 3b further decomposes this phenomenon by grouping detected reflectance photons according to the number of absorption-emission cycles they underwent. Each additional cycle shifts the spectrum incrementally toward longer wavelengths, indicating energy loss through sequential re-emission.

### 3.3. Other Distortion Effects

Scattering not only increases the probability of re-absorption but also introduces additional systematic distortions [14]. In strongly scattering media, the excitation light penetrates only a short distance into the slab geometry, localizing fluorescence generation near the illuminated surface. This spatial inhomogeneity is accounted for in our Monte Carlo simulation. In contrast, conventional integrating sphere setups, where the sample is placed inside the sphere, introduce additional mixing [16,33]. Photons strike the sample from all angles multiple times, averaging out directional effects and artificially increasing re-excitation probability. To avoid this mixing artifact and distinguish between the direction of reflectance and transmission, we positioned the sample outside the sphere during our measurements.

Scattering can also redirect photons away from fluorophores and increase the probability of their absorption by non-fluorescent components, such as the cuvette glass, solvent, or scatterers. If these effects are not distinguished, the number of true excitation events is overestimated. For example, neglecting to differentiate these absorption sites results in an even lower quantum yield of $\Phi_{f}^{\mathrm{gross}}=0.85$ for sample $c_{3}$. Our simulation framework resolves this ambiguity by modeling scattering and absorption for each material component individually, enabling accurate retrieval of $\Phi_{f}$ even in complex, highly scattering systems.

### 3.4. Quantum Yield in PDMS

In addition to the ethanol-based samples, Rhodamine 6G was also embedded in a PDMS matrix to evaluate fluorescence efficiency in a solid scattering media. The procedure is identical to that in the ethanol samples, but the optical properties of the PDMS matrix were used in the simulation. The fitted quantum yields for two concentrations $c_{a}$ and $c_{b}$ are presented in Table 2. Compared to the ethanol solutions, a decrease in $\Phi_{f}$ is observed.

The reduction in quantum yield is consistent with known solvent effects on Rhodamine dyes. Ethanol, being a polar and hydrogen-bonding solvent, stabilizes the excited state and reduces non-radiative decay [38,39]. In contrast, PDMS is non-polar and aprotic, lacking such stabilizing interactions [40]. These differences may reduce excited-state stabilization and increase the probability of non-radiative pathways, such as internal conversion [41]. Additionally, rigid matrices like PDMS have been reported to influence molecular relaxation dynamics, which could further affect $\Phi_{f}$ [40]. While these effects are plausible contributors, they are not directly measured in this study and should be interpreted with caution. These effects collectively reduce the radiative efficiency and therefore the quantum yield of the dye.

## 4. Conclusions

In this work, we presented a GPU-accelerated Monte Carlo framework for the quantitative determination of the fluorescence quantum yield in complex, highly scattering media. The spectral modeling of the fluorescence cascade was based on experimentally determined optical properties. This allowed us to correctly account for re-absorption-induced signal distortions depending on the light propagation. Explicit consideration of the experimentally applied integrating sphere geometry created a digital optical twin with which the simulation and sample could be compared spectrally resolved. This enabled a precise reconstruction of the measured emission spectrum and determination of the fluorescence quantum yield. In contrast to conventional methods for determining quantum yields in scattering media, our approach solved the complete radiative transfer equation with fluorescence term. This approach obviated the necessity for empirical correction factors. The agreement between the quantum yields of Rhodamine 6G in ethanol is obtained and the existing literature values served to confirm the reliability of the model, even in the presence of additional scatterers. The applicability of the model was further demonstrated for solid-state systems, where the embedding matrix exerted a significant influence on the radiation efficiency. Our methodology can be applied to a wide range of luminescent materials beyond Rhodamine 6G. However, achieving accurate results depends on appropriately modeling the emission probability distribution. Further research may encompass the exploration of light propagation in multilayer or anisotropic geometries, the optimization of phosphors in LEDs, and the development of models for time-resolved fluorescence processes.

## Figures and Tables

**Table 1:** Fitted quantum yields $\Phi_{f}$ for Rhodamine 6G in ethanol at three concentrations. Statistical errors are derived from the Levenberg–Marquardt fit.

Concentration	$\Phi_{f}$	Statistical Error	95% CI
$c_{1}$	0.91	±0.006	[0.90, 0.92]
$c_{2}$	0.94	±0.005	[0.93, 0.95]
$c_{3}$	0.93	±0.003	[0.92, 0.94]

**Table 2:** Fitted quantum yields $\Phi_{f}$ for Rhodamine 6G embedded in a PDMS matrix. Statistical errors are derived from the Levenberg–Marquardt fit.

Concentration	$\Phi_{f}$	Statistical Error	95% CI
$c_{a}$	0.75	±0.003	[0.74, 0.75]
$c_{b}$	0.78	±0.003	[0.78, 0.79]

## Data Availability

The data that support the findings of this study are available from the corresponding author, P.G., upon reasonable request due to privacy.
